# Supplementary material for: Quantification of subtype purity in Luminal A breast cancer predicts clinical characteristics and survival
Source: Breast Cancer Res Treat. 2023 May 20;200(2):225–35. doi: 10.1007/s10549-023-06961-9 (PMC10241706; doi:10.1007/s10549-023-06961-9)

| **Table S1.** Key features of the TCGA and METABRIC datasets | | | |
| --- | --- | --- | --- |
| *Feature* | *TCGA-BRCA* | *METABRIC* | *Notes* |
| All genes | 20,532 | 17,814 | 15,747 genes overlap |
| PAM50 genes | 50 | 47 | 47 genes overlap |
| Genomic technology | Illumina HiSeq (mRNA) | Illumina HT-12v3 (mRNA); Affymetrix SNP 6.0 array (CNA) |  |
| No. of cases | 1,081 | 1,980 | Luminal A: 505 (TCGA), 674 (METABRIC) |
| Normalization done at source | Median centering | Median centering and log transform |  |
| Normalization done by authors | Log transform | Linear transform to match mean and variance to TCGA cohort for each gene |  |

| **Table S2**. Characteristics of Luminal A breast cancers in the combined cohort (TCGA, METABRIC), stratified by quartile of negative Shannon’s Entropy subtype purity based on transcriptome | | | | | |
| --- | --- | --- | --- | --- | --- |
|  | **Q1**  n = 295 | **Q2**  n = 294 | **Q3**  n = 295 | **Q4**  n = 295 | ***P*, Q1 vs Q4**  **(p trend)** |
| Age (mean) | 63.06 | 62.04 | 60.35 | 60.03 | 0.006  (0.002) |
| ER+^a^ (%) | 97.23 | 97.93 | 98.25 | 98.93 | 0.222  (0.186) |
| PR+ (%) | 78.32 | 80.76 | 85.02 | 85.22 | 0.043  (0.013) |
| HER2+^b^ (%) | 10.82 | 8.46 | 7.63 | 4.31 | 0.005  (0.007) |
| ER+ or PR+, HER2- | 88.62 | 90.35 | 91.42 | 95.22 | 0.004  (0.008) |
| TNBC^c^ (%) | 1.20 | 0.86 | 0.40 | 0.40 | 0.373  (0.902) |
| Node positive (%) | 49.64 | 44.92 | 45.07 | 44.96 | 0.322  (0.298) |
| Stage > 1 (%) | 72.23 | 66.54 | 64.56 | 62.03 | 0.006  (0.014) |
| Tumor size > 20mm (%) | 63.73 | 58.64 | 57.97 | 50.17 | 0.001  (0.001) |
| Proliferation score^d^ (mean) | 8.94 | 8.78 | 8.58 | 8.42 | <0.001  (<0.001) |
| Recurrence score^d^ (mean) | 58.97 | 48.31 | 38.97 | 29.38 | <0.001  (<0.001) |
| Mammaprint® High-risk (%) | 24.40 | 14.97 | 7.80 | 1.02 | <0.001  (<0.001) |
| Oncotype DX® (mean) | 35.89 | 34.78 | 35.47 | 28.05 | <0.001  (<0.001) |
| Oncotype DX® High-risk (%) | 54.92 | 50.00 | 60.00 | 36.95 | <0.001  (<0.001) |
| Somatic mutations (%) |  |  |  |  |  |
| *TP53* | 17.29 | 15.93 | 7.14 | 5.08 | <0.001  (<0.001) |
| *PIK3CA* | 44.41 | 42.32 | 50.34 | 62.71 | <0.001  (<0.001) |
| *CBFB* | 3.37 | 9.15 | 3.40 | 9.49 | 0.004  (0.074) |

^a^ ER positive by immunohistochemistry; ^b^ HER2 positive by IHC or FISH; ^c^ Triple-negative by IHC; ^d^ Proliferation and Recurrence score by PAM50 genes

| **Table S3**. Characteristics of Luminal A breast cancers in the combined cohort (TCGA, METABRIC), stratified by quartile of -DRC subtype purity based on transcriptome | | | | | |
| --- | --- | --- | --- | --- | --- |
|  | **Q1**  n = 295 | **Q2**  n = 294 | **Q3**  n = 295 | **Q4**  n = 295 | ***P*, Q1 vs Q4**  **(p trend)** |
| Age (mean) | 62.32 | 61.98 | 60.78 | 60.40 | 0.072  (0.039) |
| ER+^a^ (%) | 97.60 | 98.23 | 98.60 | 97.92 | 1.000  (1.000) |
| PR+ (%) | 71.13 | 83.80 | 86.90 | 86.59 | <0.001  (<0.001) |
| HER2+^b^ (%) | 10.08 | 8.67 | 5.81 | 6.75 | 0.183  (0.190) |
| ER+ or PR+, HER2- | 89.86 | 88.89 | 94.05 | 92.80 | 0.241  (0.079) |
| TNBC^c^ (%) | 1.45 | 0.85 | 0.00 | 0.40 | 0.373  (0.084) |
| Node positive (%) | 47.90 | 44.57 | 45.75 | 46.26 | 0.741  (0.769) |
| Stage > 1 (%) | 67.03 | 66.67 | 62.11 | 69.06 | 0.659  (0.900) |
| Tumor size > 20mm (%) | 62.03 | 60.27 | 53.22 | 55.10 | 0.112  (0.032) |
| Proliferation score^d^ (mean) | 9.06 | 8.84 | 8.64 | 8.18 | <0.001  (<0.001) |
| Recurrence score^d^ (mean) | 69.17 | 51.09 | 35.86 | 19.45 | <0.001  (<0.001) |
| Mammaprint® High-risk (%) | 26.45 | 12.93 | 6.78 | 2.03 | <0.001  (<0.001) |
| Oncotype DX® (mean) | 33.85 | 37.90 | 32.38 | 30.08 | <0.001  (<0.001) |
| Oncotype DX® High-risk (%) | 51.86 | 57.14 | 47.12 | 45.76 | <0.001  (<0.001) |
| Somatic mutations (%) |  |  |  |  |  |
| *TP53* | 17.29 | 12.59 | 10.85 | 4.75 | <0.001  (<0.001) |
| *PIK3CA* | 46.78 | 44.56 | 50.85 | 57.63 | 0.011  (0.003) |
| *CBFB* | 6.10 | 4.75 | 8.16 | 6.78 | 0.867  (0.402) |

^a^ ER positive by immunohistochemistry; ^b^ HER2 positive by IHC or FISH; ^c^ Triple-negative by IHC; ^d^ Proliferation and Recurrence score by PAM50 genes

| **Table S4**. Characteristics of Luminal A breast cancers in the METABRIC cohort, stratified by tertile of pLumA subtype purity based on transcriptome | | | | |
| --- | --- | --- | --- | --- |
|  | **T1**  n = 223 | **T2**  n = 222 | **T3**  n = 229 | ***P*, T1 vs T3**  **(p trend)** |
| Age (mean) | 64.88 | 63.89 | 59.65 | <0.001  (0.001) |
| ER+^a^ (%) | 98.20 | 97.72 | 98.21 | 1.000  (1.000) |
| PR+ (%) | 64.13 | 79.73 | 86.02 | <0.001  (<0.001) |
| HER2+^b^ (%) | 7.62 | 4.51 | 1.31 | 0.001  (0.001) |
| ER+ or PR+, HER2-  and Low Proliferation (%) by AURKA | 39.00 | 78.54 | 93.89 | <0.001 (<0.001) |
| TNBC^c^ (%) | 1.30 | <1.00 | <1.00 | 0.367  (0.439) |
| Node positive (%) | 46.85 | 42.53 | 38.43 | 0.028  (0.078) |
| Grade score >= 3 | 40.00 | 24.88 | 13.02 | <0.001  (<0.001) |
| Stage > 1 (%) | 64.42 | 53.19 | 48.59 | 0.003  (0.003) |
| Tumor size > 20mm (%) | 61.88 | 48.65 | 39.04 | <0.001  (<0.001) |
| HER2 gain (%) | 17.49 | 9.46 | 6.12 | <0.001  (<0.001) |
| High Proliferation (%) AURKA | 57.14 | 20.39 | 4.17 | <0.001  (<0.001) |
| PAM50 Proliferation score^d^ (mean) | 9.08 | 9.05 | 8.99 | <0.001  (<0.001) |
| PAM50 Recurrence score^d^ (mean) | 70.40 | 54.84 | 37.48 | <0.001  (<0.001) |
| Mammaprint® High-risk (%) | 24.22 | 12.16 | 8.73 | <0.001  (<0.001) |
| Oncotype DX® (mean) | 31.92 | 25.11 | 19.94 | <0.001  (<0.001) |
| Oncotype DX® High-risk (%) | 50.22 | 18.92 | 4.37 | <0.001  (<0.001) |
| Somatic mutations (%) |  |  |  |  |
| *TP53* | 20.18 | 10.36 | 6.99 | <0.001  (<0.001) |
| *PIK3CA* | 41.26 | 58.11 | 71.62 | <0.001  (<0.001) |
| *CBFB* | 3.14 | 10.36 | 12.23 | <0.001  (<0.001) |

^a^ ER positive by immunohistochemistry; ^b^ HER2 positive by IHC or FISH; ^c^ Triple-negative by IHC; ^d^ Proliferation and Recurrence score by PAM50 genes

| **Table S5**. Characteristics of Luminal A breast cancers in the TCGA cohort, stratified by tertile of pLumA subtype purity based on transcriptome | | | | |
| --- | --- | --- | --- | --- |
|  | **T1**  n = 167 | **T2**  n = 166 | **T3**  n = 172 | ***P*, T1 vs T3**  **(p trend)** |
| Age (mean) | 58.43 | 59.87 | 60.19 | 0.064  (0.218) |
| ER+^a^ (%) | 97.58 | 98.14 | 98.73 | 1.000  (0.699) |
| PR+ (%) | 87.66 | 90.24 | 92.63 | 0.479  (0.330) |
| HER2+^b^ (%) | 23.63 | 17.43 | 11.12 | 0.014  (0.034) |
| ER+ or PR+, HER2- | 76.36 | 80.73 | 88.70 | 0.021 (0.041) |
| TNBC^c^ (%) | 1.00 | 1.00 | 0.00 | 1.000  (1.000) |
| Node positive (%) | 54.49 | 49.65 | 51.28 | 0.265  (0.670) |
| Stage > 1 (%) | 79.63 | 79.87 | 75.89 | 0.431  (0.315) |
| Tumor size > 20mm (%) | 72.09 | 67.47 | 64.67 | 0.131  (0.109) |
| PAM50 Proliferation score^d^ (mean) | 8.45 | 8.18 | 7.95 | <0.001  (<0.001) |
| PAM50 Recurrence score^d^ (mean) | 35.09 | 29.27 | 26.56 | <0.001  (<0.001) |
| Mammaprint® High-risk (%) | 19.16 | 10.24 | 5.81 | <0.001  (<0.001) |
| Oncotype DX® (mean) | 49.95 | 43.64 | 38.99 | <0.001  (<0.001) |
| Oncotype DX® High-risk (%) | 89.22 | 88.55 | 78.49 | <0.001  (<0.001) |
| Mutational load (median) | 26.50 | 23.00 | 22.00 | 0.449  (0.432) |
| MATH score (mean) | 0.363 | 0.382 | 0.381 | 0.232  (0.323) |
| Somatic mutations (%) |  |  |  |  |
| *TP53* | 16.17 | 11.45 | 2.33 | <0.001  (<0.001) |
| *PIK3CA* | 31.74 | 41.57 | 47.09 | <0.001  (<0.001) |
| *CBFB* | 2.99 | 3.01 | 4.65 | 0.574  (0.640) |

^a^ ER positive by immunohistochemistry; ^b^ HER2 positive by IHC or FISH; ^c^ Triple-negative by IHC; ^d^ Proliferation and Recurrence score by PAM50 genes


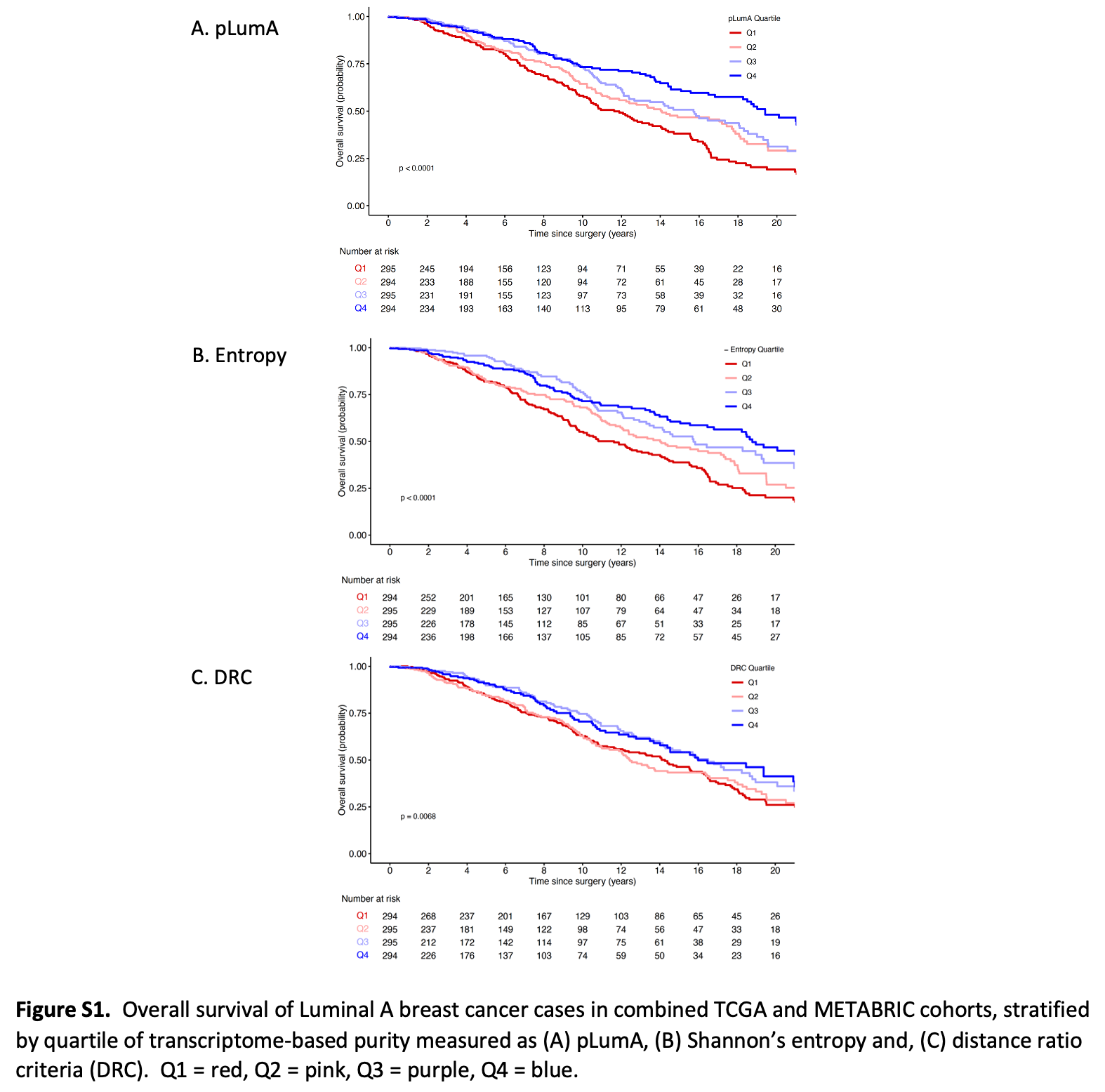


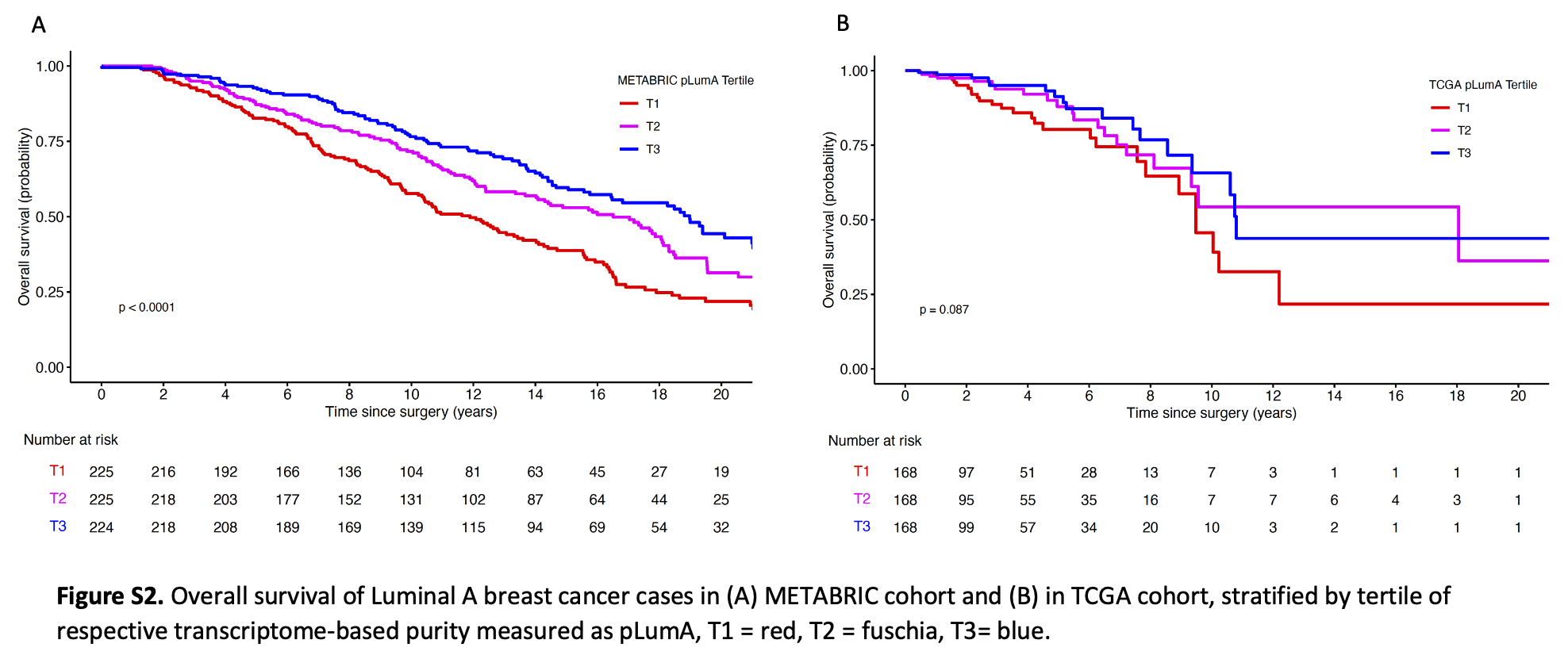


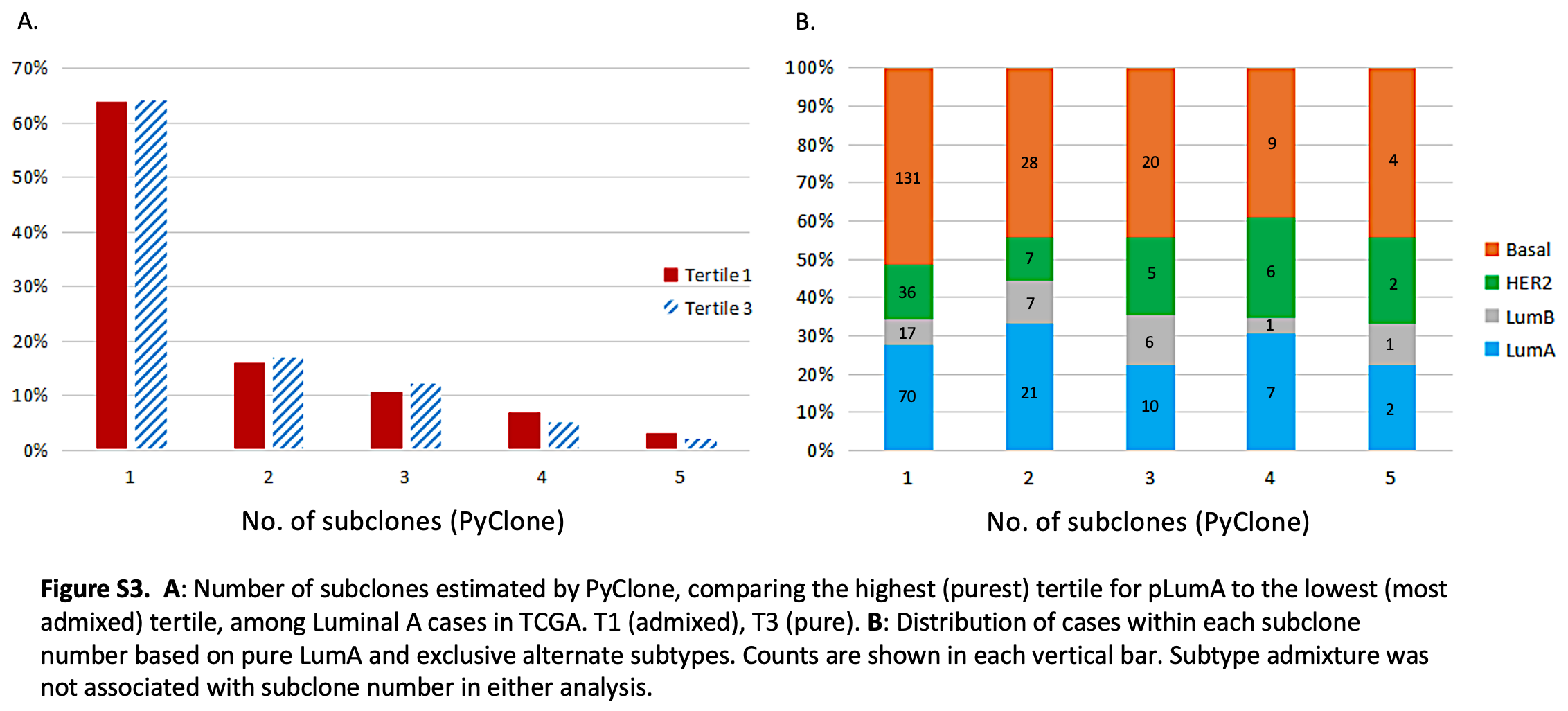

Supplement: Supplementary file 1 — Supplementary file1 (DOCX 1004 kb) [file 10549_2023_6961_MOESM1_ESM.docx]
